# From fatigue to physiology: Submaximal 2‐day cardiopulmonary exercise test and emerging standards in long COVID

**DOI:** 10.1113/EP092958

**Published:** 2025-06-18

**Authors:** Michael G. Risbano

**Affiliations:** ^1^ University of Pittsburgh Pittsburgh Pennsylvania USA

**Keywords:** CPET, iCPET, Long COVID, PASC, Post‐Exertional Malaise, Post‐Exertional Symptom Exacerbation

1

Persistent fatigue and activity intolerance in post‐acute sequelae of COVID‐19 (PASC) presents one of the most elusive diagnostic and therapeutic challenges in contemporary medicine. Post‐exertional symptom exacerbation (PESE), a worsening of symptoms following minimal exertion, mirrors the hallmark post‐exertional malaise (PEM) long described in myalgic encephalomyelitis/chronic fatigue syndrome (ME/CFS), which may share post‐infectious origins (Blomberg et al., 2018). While widely recognized by patients, PESE remains physiologically under‐defined. The recent analysis of PESE in PASC patients in this issue of *Experimental Physiology* by Thomas et al. provides critical progress toward resolving this gap (Thomas et al., 2025).

Thomas and colleagues utilized a 2‐day submaximal stepwise incremental cardiopulmonary exercise test (CPET) protocol in 68 individuals with PASC, where Day 2 was intended to identify physiological changes elicited by Day 1 exertion. The authors observed significant declines in key physiological parameters at the first ventilatory threshold (VT₁), with no notable differences at peak‐level exercise. They noted a decrease of 6.8% in oxygen consumption (${\dot{V}}_{O_{2}}$) over a 24‐h period. Similarly, O₂ pulse declined by 8.5%, and $P_{\mathrm{ETC}O_{2}}$ decreased by 2.6%. These changes were accompanied by a lower workload, a modest reduction in minute ventilation (*V̇*_E_), and a small decline in carbon dioxide production (${\dot{V}}_{CO_{2}}$).

While each of these changes is modest in isolation, the consistent decline across multiple physiological domains may, however, reflect impaired submaximal exercise tolerance, or effort limitation on Day 2. Given the substantial inter‐individual variability that ranged from ∼± 9% for end‐tidal carbon dioxide pressure ($P_{\mathrm{ETC}O_{2}}$) to over ±22% for O₂ pulse and ${\dot{V}}_{O_{2}}$, these findings may fall within the range of potential measurement noise. This raises an important question: what degree of change in cardiopulmonary exercise parameters performed over a 24‐h interval constitutes true physiological deterioration versus normal biological or even technical fluctuation? In the absence of standardized reproducibility thresholds or normative datasets for 2‐day CPET protocols, particularly in symptomatic long COVID populations, interpretation of small absolute changes remains challenging and context dependent. The authors concluded that this profile of impairment at VT_1_ identifies diminished O_2_ uptake and delivery without overt ventilatory or chronotropic limitation, suggesting impairments in central and peripheral O_2_ transport. While Thomas et al. employed non‐invasive measures, the findings align well with the pathophysiology identified by invasive CPET (iCPET) studies (Risbano et al., 2023).

Our work evaluating long COVID subjects with iCPET (Risbano et al., 2023) has identified distinct physiological endotypes including preload failure and impaired systemic O_2_ extraction. Preload failure consists of low biventricular filling pressures with reduced cardiac output in the upright position (with normal left ventricular function) at peak exercise and at submaximal effort (Fakhri et al., 2025; Oldham et al., 2016). This phenotype is mechanistically linked to impaired stroke volume augmentation and can overlap with inadequate peripheral O₂ utilization.

Although not derived from iCPET, the findings reported by Thomas et al. (2025) may be consistent with this physiological framework. Oxygen pulse, which serves as a rough surrogate for stroke volume and peripheral oxygen extraction efficiency, and $P_{\mathrm{ETC}O_{2}}$, a complex measure reflecting the combined effects of pulmonary perfusion, alveolar ventilation and cardiac output, both declined on Day 2. The combination of preserved heart rate, reduced O_2_ pulse and a stable ${\dot{V}}_{E}/{\dot{V}}_{CO_{2}}$ suggests impaired central delivery or diminished peripheral extraction, or reduced mitochondrial uptake, rather than ventilatory inefficiency or generalized deconditioning. Although non‐invasive CPET cannot localize physiological dysfunction, the patterns described could be interpreted through the lens of preload failure and peripheral metabolic dysregulation.

Gattoni et al. used a 2‐day peak‐exercise CPET design in 15 participants enriched for PEM, and identified a reduction in ${\dot{V}}_{E}/{\dot{V}}_{CO_{2}}$ at the gas exchange threshold (GET), but no other significant physiological changes at GET or peak exercise (Gattoni et al., 2025). The use of maximal testing may have obscured submaximal abnormalities relevant to PESE, as compensatory mechanisms at peak effort could mask deficits in O_2_ delivery or extraction. In contrast, the submaximal approach used by Thomas et al. (2025) may better capture early physiological deterioration under conditions more reflective of daily activity. The absence of significant findings in Gattoni's study may also reflect limited power, high inter‐individual variability, and methodological factors such as variability in threshold identification (GET or VT_1_) or the use of transcutaneous rather than end‐tidal CO₂. Studies like those by Thomas and Gattoni, while methodologically distinct, are helping define the clinical physiology of PESE.

iCPET is beneficial in identifying reduced biatrial filling pressures, cardiac output augmentation and abnormal oxygen extraction, but this methodology is resource‐intensive and requires specialized infrastructure to complete these studies. Thomas et al. (2025) provide a scalable framework; if coupled with careful interpretation of the surrogate markers O_2_ pulse and $P_{\mathrm{ETC}O_{2}}$, it could serve as a useful screening or phenotyping tool for preload failure and impaired O_2_ extraction in PESE patients.

Future directions should focus on linking level 1 CPET patterns with iCPET‐defined physiology, validating surrogate markers of preload failure and impaired O_2_ extraction, and establishing reproducibility thresholds for submaximal protocols. As small but consistent declines in ${\dot{V}}_{O_{2}}$, O₂ pulse and workload emerge across 2‐day testing, the need to define what constitutes meaningful change becomes clear. Post‐viral fatigue is not only measurable, but reproducible and biologically grounded. Thomas et al. (2025) advance this understanding, and we are building on their work to improve evaluation and care for patients with long COVID and other post‐infectious syndromes.

## AUTHOR CONTRIBUTIONS

Sole author.

## CONFLICT OF INTEREST

None declared.

## References

[eph13918-bib-0001] Blomberg, J. , Gottfries, C. G. , Elfaitouri, A. , Rizwan, M. , & Rosen, A. (2018). Infection elicited autoimmunity and myalgic encephalomyelitis/chronic fatigue syndrome: An explanatory model. Frontiers in Immunology, 9, 229. 29497420 10.3389/fimmu.2018.00229 PMC5818468

[eph13918-bib-0002] Fakhri, S. , Campedelli, L. , & Risbano, M. G. (2025). Hemodynamic responses at anaerobic threshold during exercise in preload insufficiency. European Journal of Clinical Investigation, 55(2), e14343. 39528407 10.1111/eci.14343 PMC11744911

[eph13918-bib-0003] Gattoni, C. , Abbasi, A. , Ferguson, C. , Lanks, C. W. , Decato, T. W. , Rossiter, H. B. , Casaburi, R. , & Stringer, W. W. (2025). Two‐day cardiopulmonary exercise testing in long COVID post‐exertional malaise diagnosis. Respiratory Physiology & Neurobiology, 331, 104362. 39490617 10.1016/j.resp.2024.104362

[eph13918-bib-0004] Oldham, W. M. , Lewis, G. D. , Opotowsky, A. R. , Waxman, A. B. , & Systrom, D. M. (2016). Unexplained exertional dyspnea caused by low ventricular filling pressures: Results from clinical invasive cardiopulmonary exercise testing. Pulmonary Circulation, 6(1), 55–62. 27162614 10.1086/685054 PMC4860548

[eph13918-bib-0005] Risbano, M. G. , Kliment, C. R. , Dunlap, D. G. , Koch, C. , Nouraie, S. M. , Sciurba, F. C. , & Morris, A. (2023). Invasive cardiopulmonary exercise testing identifies distinct physiologic endotypes in post‐acute sequelae of SARS‐CoV‐2 infection. Chest Pulmonary, 1(3), 1–16. 10.1016/j.chpulm.2023.100010 PMC13418082 42548372

[eph13918-bib-0006] Thomas, C. , Kudiersky, N. , Ansdell, P. , Ashton, R. , Brown, C. , Bewick, T. , Carr, J. , Hume, E. , Spillane, P. , Pastorio, E. , Owen, R. , Wilkinson, T. M. , McNeil‐Angopa, E. , Parkington, T. , Arena, R. , Ozemek, C. , Formenti, F. , Veluswamy, S. K. , Gururaj, R. , & Faghy, M. A. (2025). Submaximal 2‐day cardiopulmonary exercise testing to assess exercise capacity and post‐exertional symptom exacerbation in people with Long COVID. Experimental Physiology, 10.1113/EP092508 PMC12857497 40515424

